# Mechanisms for autophagy modulation by isoprenoid biosynthetic pathway inhibitors in multiple myeloma cells

**DOI:** 10.18632/oncotarget.6365

**Published:** 2015-11-22

**Authors:** Kaitlyn M. Dykstra, Cheryl Allen, Ella J. Born, Huaxiang Tong, Sarah A. Holstein

**Affiliations:** ^1^ Department of Medicine, Roswell Park Cancer Institute, Buffalo, NY, USA; ^2^ Department of Internal Medicine, University of Iowa, Iowa City, IA, USA; ^3^ Penn State Hershey Cancer Institute, Hershey, PA, USA; ^4^ Department of Immunology, Roswell Park Cancer Institute, Buffalo, NY, USA

**Keywords:** myeloma, RabGTPase, autophagy, prenylation, isoprenoid

## Abstract

Multiple myeloma (MM) is characterized by the production of monoclonal protein (MP). We have shown previously that disruption of the isoprenoid biosynthetic pathway (IBP) causes a block in MP secretion through a disruption of Rab GTPase activity, leading to an enhanced unfolded protein response and subsequent apoptosis in MM cells. Autophagy is induced by cellular stressors including nutrient deprivation and ER stress. IBP inhibitors have been shown to have disparate effects on autophagy. Here we define the mechanisms underlying the differential effects of IBP inhibitors on autophagic flux in MM cells utilizing specific pharmacological inhibitors. We demonstrate that IBP inhibition induces a net increase in autophagy as a consequence of disruption of isoprenoid biosynthesis which is not recapitulated by direct geranylgeranyl transferase inhibition. IBP inhibitor-induced autophagy is a cellular defense mechanism as treatment with the autophagy inhibitor bafilomycin A1 enhances the cytotoxic effects of GGPP depletion, but not geranylgeranyl transferase inhibition. Immunofluorescence microscopy studies revealed that IBP inhibitors disrupt ER to Golgi trafficking of monoclonal light chain protein and that this protein is not a substrate for alternative degradative pathways such as aggresomes and autophagosomes. These studies support further development of specific GGTase II inhibitors as anti-myeloma agents.

## INTRODUCTION

**Figure 1:**
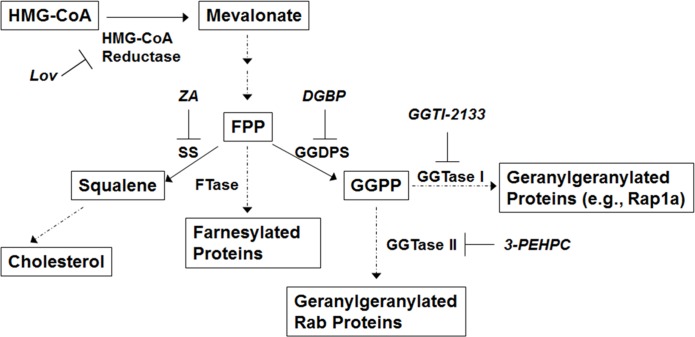
The isoprenoid biosynthetic pathway Relevant enzymes and specific inhibitors are shown. Abbreviations: farnesyl pyrophosphate (FPP), geranylgeranyl pyrophosphate (GGPP), lovastatin (Lov), squalene synthase (SS), zaragozic acid (ZA).

Multiple myeloma is a plasma cell malignancy characterized by the production of monoclonal protein. There has been interest in developing therapeutic strategies which exploit the underlying physiology of the highly secretory plasma cells. We have previously demonstrated that agents which disrupt geranylgeranylation of Rab GTPases, either through depletion of the isoprenoid donor geranylgeranyl diphosphate (GGPP) or direct inhibition of the responsible prenyl transferase (geranylgeranyl transferase II (GGTase II)) (Figure 1), interfere with intracellular monoclonal protein trafficking. [1] This results in an accumulation of monoclonal protein within the cell, induction of ER stress, the unfolded protein response pathway (UPR) and apoptosis. [1, 2]

The primary mechanisms through which protein can be degraded involve the proteasome, aggresomes, and autophagy and these pathways are integrally linked. Autophagosomes, which are double-membrane vesicles, engulf cytosolic proteins and organelles, and then fuse with lysosomes. Autophagy occurs at a basal rate in all cells and may be induced by stressors such as nutrient deprivation or stimulation of the UPR via ER-stress inducers. [3, 4] Of note, several Rabs have been identified as playing roles in autophagy, including Rab32 and Rab33, [5, 6] which are positive regulators of elongation of the isolation membrane, and Rab7, which is involved with autophagosome fusion with lysosomes. [7, 8] There have been several reports demonstrating the role for autophagy in clearing toxic aggregated proteins such as mutant huntingtin and ataxin-1. [9-11] Whether autophagy plays a role in clearing aggregated monoclonal protein is unknown.

Thapsigargin (an ER-stress inducer), rapamycin (an mTOR inhibitor), and bortezomib (a proteasome inhibitor) have all been shown to induce markers of autophagy in myeloma cells. [12] The combination of bortezomib and the autophagy inhibitor chloroquine [12], yielded antagonistic effects on cell survival, yet the combination of bortezomib and bafilomycin A1, an agent which is also considered to be an inhibitor of late stage events in autophagy, induced a synergistic cytotoxic effect in myeloma cells. [13] A phase I study has been conducted in patients with relapsed/refractory myeloma assessing the combination of hydroxychloroquine with bortezomib. [14] Proteasome inhibitor resistance has been associated with increased levels of autophagy. [15] These studies suggest a complex relationship between the proteasome degradation pathway, ER stress, and autophagy in malignant plasma cells.

Disparate effects on autophagy have been observed following treatment of cells with agents which interfere with the isoprenoid biosynthetic pathway (IBP) (Figure 1). This is likely a consequence of differences in cell types as well as differences amongst the IBP inhibitors. Wojtkowiak et al., demonstrated that the combination of a HMG-CoA reductase inhibitor (lovastatin) with a farnesyl transferase inhibitor (FTI-1) interfered with the completion of the autophagic cycle in malignant peripheral nerve sheath tumor cells. [16] Alternatively, statins have been shown to induce autophagy in a variety of different cell types, including airway mesenchymal cells, [17] vascular smooth muscle cells, [18] and lymphoma cells. [19] Wasko et al. demonstrated that agents which inhibit either farnesyl diphosphate synthase (FDPS) or GGPP synthase (GGDPS) induce autophagy in prostate cancer cells in a manner which appeared to be dependent on impairment of geranylgeranylation of GGTase II substrates, Rab GTPases. [20] It is still an open question, however, as to whether autophagy modulation by IBP inhibitors is due to feedback mechanisms induced by the depletion of isoprenoid levels or the disruption of the function of prenylated substrates. Additionally, little is known regarding the effects of these agents on autophagy in myeloma cells.

Given the importance of Rabs in intracellular trafficking processes and autophagy, we hypothesized that specifically targeting Rab prenylation without disrupting isoprenoid levels would not only cause an accumulation of intracellular protein which could trigger autophagy, but also could disrupt completion of autophagy, an innate pro-survival mechanism for dealing with aggregated protein. Here we define the effects of IBP and GGTase II inhibitors on autophagy in myeloma cells as well as explore the fate of accumulated monoclonal protein.

## RESULTS

### Isoprenoid biosynthetic pathway inhibitors induce increases in LC3-II levels

**Figure 2:**
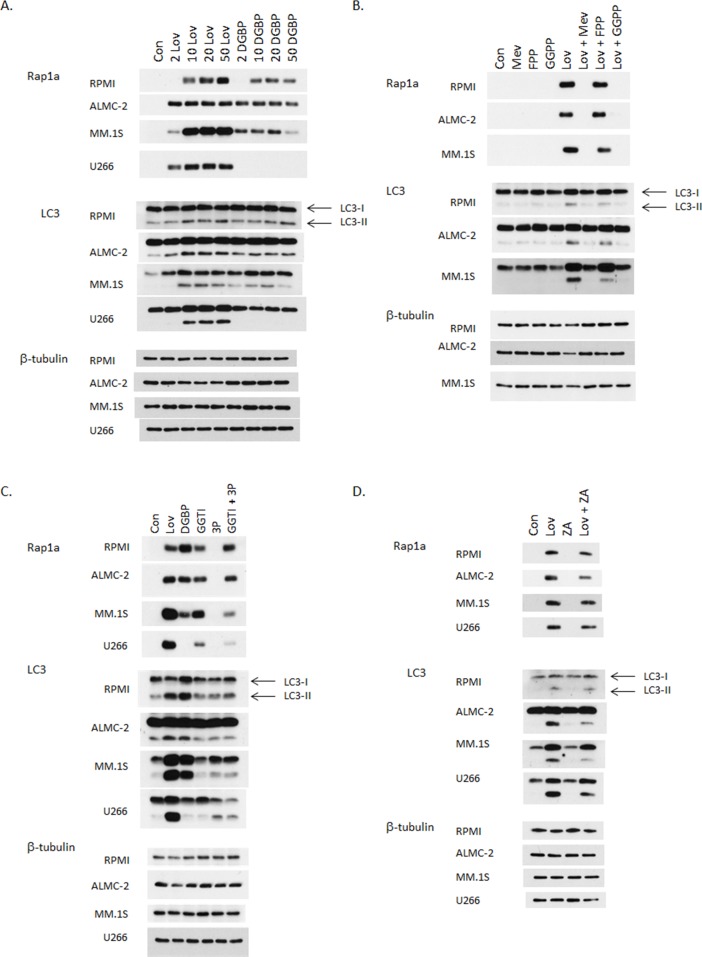
Inhibition of the isoprenoid biosynthetic pathway induces an increase in LC3-II levels in myeloma cells Immunoblot analysis for Rap1a (unmodified protein only), LC3 and β-tubulin (loading control) is shown. The gels are representative of 2-4 independent experiments. **A**. RPMI-8226, ALMC-2, MM.1S, and U266 cells were incubated for 48 hours in the presence or absence of lovastatin (*Lov*, 2-50 μM) or DGBP (2-50 μM). **B**. RPMI-8226, ALMC-2 and MM.1S cells were incubated for 48 hours in the presence of 10 μM lovastatin (*Lov*) and/or mevalonate (*Mev*, 5 mM), FPP (10 μM), or GGPP (10 μM) for 48 hrs. **C**. RPMI-8226, ALMC-2, MM.1S, and U266 cells were incubated for 48 hours in the presence or absence of 10 μM lovastatin (*Lov*), 10 μM DGBP, 10 μM GGTI-2133 (*GGTI*), 10 mM 3-PEHPC (*3P*), or the combination of 10 μM GGTI-2133 and 10 mM 3-PEHPC (*GGTI + 3P*). **D**. Cells were incubated for 48 hours in the presence or absence of 20 μM lovastatin (*Lov*) and/or zaragozic acid A (*ZA*) (10 μM for RPMI-8226 and 50 μM for all other cell lines).

During autophagy LC3-I is lipidated to yield LC3-II. Detection of LC3-II via immunoblot analysis is a well-established methodology by which to assess autophagy. [21] We first investigated the effects of two IBP inhibitors on levels of LC3-II levels in myeloma cells. As shown in Figure 2A, human myeloma and AL amyloid cells were treated with increasing concentrations of lovastatin, an HMG-CoA reductase inhibitor, or digeranyl bisphosphonate (DGBP), a GGDPS inhibitor for 48 hrs. Immunoblots depicting unmodified Rap1a levels are shown to demonstrate successful disruption of protein geranylgeranylation. Rap1a is a substrate of geranylgeranyl transferase I (GGTase I) and the antibody used detects only unmodified Rap1a. In addition, ELISA analysis of cell lysate (Supplementary Figure 1) demonstrates an intracellular accumulation of light chain protein, which is a direct consequence of disruption of Rab geranylgeranylation. [1] That lovastatin depletes both FPP and GGPP while DGBP depletes GGPP but increases FPP, was confirmed by measuring intracellular FPP and GGPP levels (Supplementary Figure 2). Consistent with our previous studies, [22] the U266 cell line is completely resistant to DGBP, providing an important internal control for these experiments. An increase in LC3-II levels was observed with lovastatin treatment in all tested cell lines as well as with DGBP with the exception of the U266 cell line. This increase in LC3-II was time-dependent, appearing after the first signs of disruption of protein geranylgeranylation (Supplementary Figure 3).

As both lovastatin and DGBP deplete cells of GGPP, it was hypothesized that the observed changes in markers of autophagy were a consequence of disruption of protein geranylgeranylation by virtue of GGPP depletion. Studies were performed in which cells were treated with both lovastatin and either mevalonate, FPP, or GGPP. As shown in Figure 2B, mevalonate and GGPP, but not FPP completely prevents lovastatin-induced disruption of protein geranylgeranylation as evidenced by changes in unmodified Rap1a levels and intracellular light chain levels (Supplementary Figure 4). While mevalonate and GGPP completely prevent the lovastatin-induced increase in LC3-II levels, FPP only partially abrogates the effects of lovastatin on LC3-II levels while having no effect on markers of geranylgeranylation (Figure 2B, Supplementary Figure 5). In aggregate these results demonstrate that disruption of protein geranylgeranylation alters a key marker of autophagy and provides evidence that other isoprenoid-related processes are also involved in regulating autophagic flux.

To further discriminate between the effects of isoprenoid depletion versus the effects of inhibiting protein geranylgeranylation on LC3-II levels, studies were performed in which cells were treated with either the IBP inhibitors (lovastatin or DGBP) or specific GGTase I (GGTI-2133) and/or GGTase II (3-PEHPC [23]) inhibitors. We have previously demonstrated that increases in intracellular light chain occur as a consequence of disruption of Rab protein prenylation either by depleting GGPP (lovastatin/DGBP) or directly inhibiting GGTase II (3-PEHPC), but not GGTase I (GGTI-2133). [1, 2] As expected, the GGTase I inhibitor, but not the GGTase II inhibitor, disrupts Rap1a geranylgeranylation (Figure 2C) while the GGTase II inhibitor, but not the GGTase I inhibitor, disrupts Rab geranylgeranylation as evidenced by intracellular accumulation of light chain and decreases in membrane-bound Rab (Supplementary Figure 6A-6B). The GGTase I inhibitor had minimal effects on LC3-II levels in the tested cell lines. The GGTase II inhibitor, either alone or in combination also induced only minimal increases in LC3-II levels, with GGTase II inhibitor-mediated increases in LC3-II being more prominent in the MM.1S and U266 cell lines. Notably the combination of the two GGTase inhibitors did not recapitulate the effects of the IBP inhibitors on LC3-II levels, despite leading to similar increases in intracellular light chain levels, which we have previously shown to correlate with the amount of RabGTPase prenylation inhibition. [1] Treatment with a specific farnesyl transferase inhibitor did not alter LC3-II levels (data not shown). Finally, the effects of directly inhibiting sterol synthesis were investigated using zaragozic acid A, a specific inhibitor of squalene synthase. [24] As shown in Figure 2D, zaragozic acid A did not increase LC3-II levels. However, the addition of zaragozic acid A to lovastatin did partially abrogate lovastatin's effects, consistent with the increased availability of FPP for non-sterol processes. In aggregate, these data support the hypothesis that disruption of geranylgeranylation is not fully responsible for the observed effects of the inhibitors and that perturbation of non-sterol isoprenoid levels affects autophagy.

### Isoprenoid biosynthetic pathway inhibitors have disparate effects on autophagic flux

**Figure 3:**
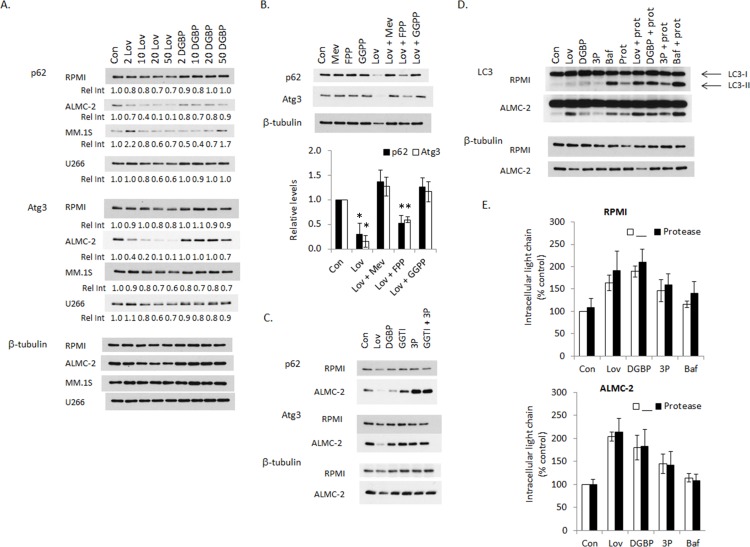
Effects of isoprenoid biosynthetic pathway inhibitors on autophagic flux **A**. RPMI-8226, ALMC-2, MM.1S, and U266 cells were incubated for 48 hours in the presence or absence of lovastatin (*Lov*, 2-50 μM) or DGBP (2-50 μM). Immunoblot analysis of p62, Atg3 and β-tubulin (loading control) is shown. The relative intensity (*Rel Int)* values underneath the p62 and Atg3 blots represent relative levels as determined by densitometric analysis of Atg3 or p62 (relative to tubulin) of treated cells compared to control cells. The gels are representative of 3-4 independent experiments. **B**. ALMC-2 cells were incubated for 48 hours in the presence of 10 μM lovastatin (*Lov*) and/or mevalonate (*Mev*, 5 mM), FPP (10 μM), or GGPP (10 μM) for 48 hrs. Densitometric analysis of p62 or Atg3 levels (normalized to β-tubulin levels) for the combination treatments normalized to untreated cells (control) is shown. Data are displayed as average +/− standard deviation (n=3 independent experiments). * denotes p-value <0.05 from two-sided t-testing. **C**. RPMI-8226 and ALMC-2 cells were incubated for 48 hours in the presence or absence of 10 μM lovastatin (*Lov*), 10 μM DGBP, 10 μM GGTI-2133 (*GGTI*), 10 mM 3-PEHPC (*3P*), or the combination of 10 μM GGTI-2133 and 10 mM 3-PEHPC (*GGTI + 3P*). Immunoblot analysis of p62, Atg3 and β-tubulin (loading control) is shown. The gels are representative of 3-4 independent experiments. **D**. RPMI-8226 and ALMC-2 cells were incubated for 48 hours in the presence or absence of 10 μM lovastatin (*Lov*), 10 μM DGBP, 10 mM 3-PEHPC (*3P*), or 5 nM bafilomycin A1 (*Baf*) with or without protease inhibitors (10 μg/mL E64d and pepstatin A, *prot*). Immunoblot analysis of LC3 and β-tubulin (loading control) is shown. The gels are representative of 3 independent experiments. **E**. Intracellular lambda light chain levels were measured via ELISA. Data are expressed as a percentage of control (mean +/− standard deviation of 3 independent experiments). Two-sided t-testing was performed to compare the IBP inhibitor alone vs. in combination with the protease inhibitors. No significant differences between the treatment groups were observed.

The ubiquitin-binding scaffold protein p62 binds LC3 and is degraded via autophagy, therefore p62 levels decrease with induction of autophagy and increase when autophagy is disrupted. [25, 26] Immunoblots for p62 demonstrated a concentration-dependent decrease in levels with lovastatin, most prominently observed in the ALMC-2 cells, with minimal changes in levels in the presence of DGBP (Figure 3A). These results suggested that the predominant effect of lovastatin is to increase autophagosome turnover. The effects of these agents on other autophagy components were also examined. Interestingly, similar to p62, lovastatin induces a concentration-dependent decrease in Atg3 levels in all tested cell lines, however the functional relevance of this is unknown. Levels of several other enzymes associated with autophagy (Atg5, Atg7, LAMP-1) are not affected by either IBP inhibitor (Supplementary Figure 7). As the effects of lovastatin on p62 and Atg3 levels were most prominent in the ALMC-2 cells, this line was chosen to examine the consequences of co-incubation with isoprenoids. As shown in Figure 3B, mevalonate and GGPP completely prevent the lovastatin-induced decrease in p62 and Atg3 levels while FPP only partially prevents this effect, consistent with the effects observed with LC3-II (Figure 2B). The GGTase II inhibitor markedly increased p62 levels in the ALMC-2 cells (Figure 3C), suggesting that the predominant effect of inhibition of Rab geranylgeranylation is to inhibit autophagy. Atg3 levels were not altered by GGTase inhibitor treatment in either cell line.

To further assess the effects of the IBP inhibitors on autophagic flux, studies were performed with protease inhibitors (E64d and pepstatin A) (Figure 3D). An increase in LC3-II may be due either to an increase in autophagosomes as a consequence of induction of autophagy or may represent an inability of autophagy to be completed as a consequence of inhibition of the later stages of autophagy. Typically it is predicted that if an agent induces autophagic flux, then the addition of the protease inhibitors will lead to a further increase in LC3-II levels while if an agent disrupts autophagic flux, then LC3-II levels should not significantly change. [21] Treatment with the protease inhibitors alone resulted in an increase in LC3-II levels. As a positive control, the late-stage autophagy inhibitor bafilomycin A1 was used and an increase in LC3-II levels was observed. The combination of each IBP inhibitor with the protease inhibitors resulted in a further increase in LC3-II levels with the most marked effects occurring with lovastatin and DGBP in the RPMI-8226 cells and lovastatin in the ALMC-2 cells. While this would suggest that these agents are primarily inducing autophagic flux, it should be noted that the addition of the protease inhibitors to bafilomycin A1 also further increased LC3-II levels, indicating that if agents are used which disrupt autophagy at different stages, there can be a further increase in LC3-II. Notably, the addition of protease inhibitors to the IBP inhibitors did not alter intracellular light chain levels compared with IBP inhibitors alone (Figure 3E). These results suggested that accumulated intracellular light chain induced by the IBP inhibitors may not be a substrate for autophagic degradation.

### IBP inhibitor-induced cell death is enhanced in the presence of autophagy inhibition

**Figure 4:**
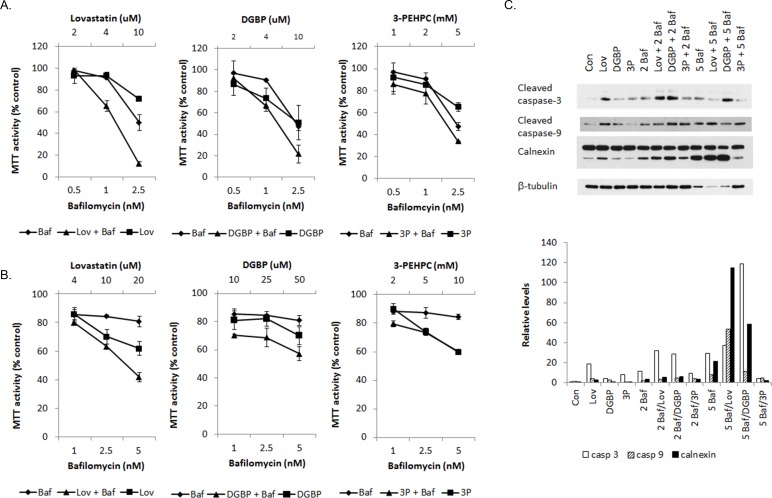
The combination of isoprenoid biosynthetic pathway inhibitors and the late stage autophagy inhibitor bafilomycin A results in increased myeloma cell death MTT cytotoxicity assays were performed with RPMI-8226 **A**. and MM.1S **B**. cells treated with bafilomycin (*Baf*) and/or lovastatin *(Lov*), DGBP, or 3-PEHPC for 48 hrs. Data are expressed as percentage of control (mean +/− standard deviation of 2 independent experiments). **C**. RPMI-8226 cells were incubated for 48 hours in the presence or absence of 10 μM lovastatin (*Lov*), 10 μM DGBP, 10 mM 3-PEHPC (*3P*) with or without 2 nM bafilomycin A1 (2 *Baf*) or 5 nM bafilomycin A1 (*5 Baf*). Immunoblot analysis of cleaved caspase-3,cleaved caspase-9, calnexin and β-tubulin (loading control) is shown. The gels are representative of 3 independent experiments. Densitometric analysis of the cleaved caspase -3, cleaved caspase-9, and cleaved calnexin relative to β-tubulin levels is shown.

We have previously demonstrated that select IBP inhibitors induce myeloma cell death in part through ER-stress related cell death pathways as a consequence of disruption of protein trafficking. [1, 2] As our studies revealed that both depletion of isoprenoids and direct inhibition of Rab geranylgeranylation alter autophagic processes, we next investigated the effects of combining the IBP inhibitors with autophagy inhibitors on myeloma cell death. As shown in Figure 4A-4B, in both RPMI-8226 and MM.1S cells, the addition of bafilomycin A1 to lovastatin or DGBP resulted in synergistic effects on cytotoxicity while minimal effects were observed with the addition of bafilomycin A1 to the GGTase II inhibitor 3-PEHPC (Supplementary Table 3). Consistent with the cytotoxicity assays, immunoblot analysis of caspase-3, caspase-9 cleavage and calnexin cleavage demonstrated enhanced induction of apoptosis when the IBP inhibitors (lovastatin and DGBP) were combined with bafilomycin A1 (Figure 4C). Enhancement of apoptosis was also seen using Annexin/PI flow cytometry with the higher concentration combinations of bafilomycin A1 with lovastatin and DGBP but not with 3-PEHPC (Supplementary Figure 8). Interestingly, the addition of bafilomycin A1 to lovastatin increased the intracellular accumulation of light chain, however this effect was not observed with either DGBP or 3-PEHPC or in the ALMC-2 cells (Supplementary Figure 9A). The addition of 3-methyladenine (3-MA), an inhibitor of earlier stages of autophagy, was either additive or antagonistic with respect to the IBP inhibitors and did not alter lovastatin-induced accumulation of intracellular light chain (Supplementary Figures 10 and 9B, Supplementary Table 3).

### IBP inhibitors block ER to Golgi trafficking of light chain

**Figure 5:**
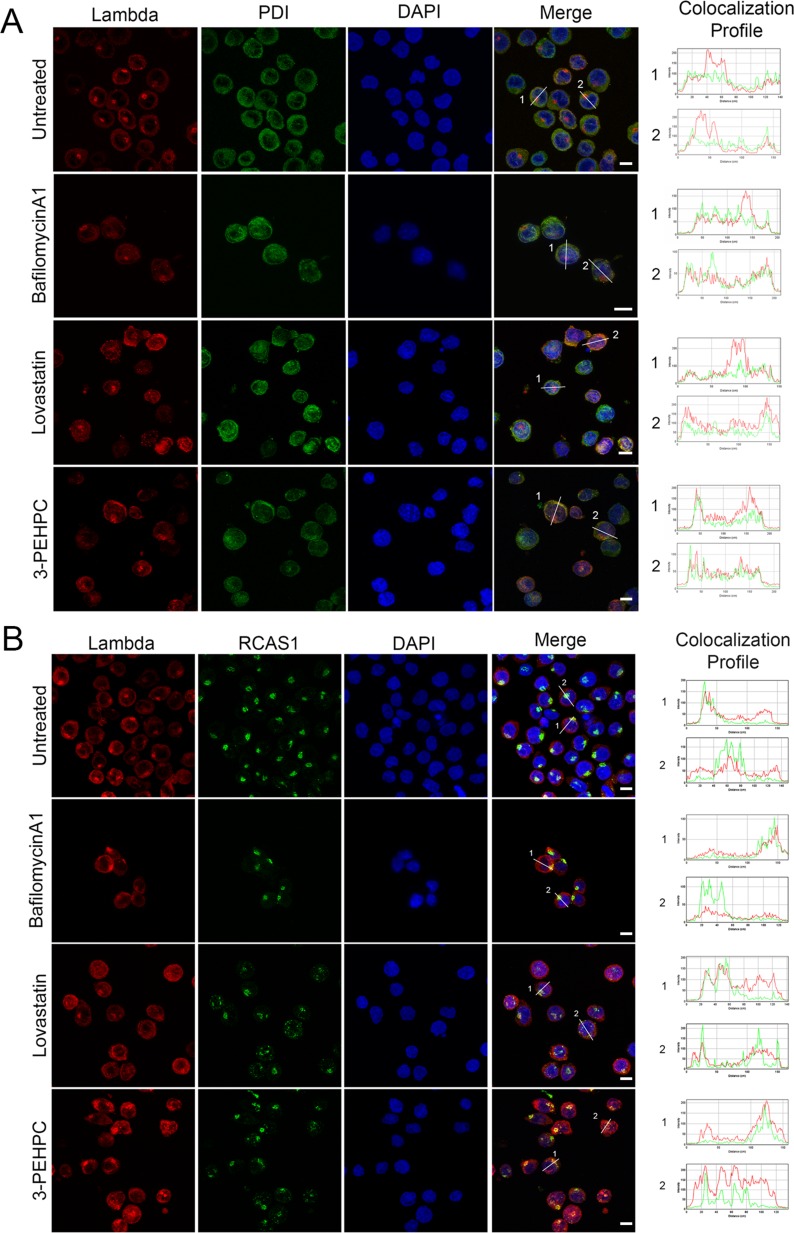
IBP inhibitors block ER to Golgi trafficking of light chain RPMI-8226 cells were incubated with 2 nM bafilomycinA1, 10 μM lovastatin, or 5 mM 3-PEHPC for 48 hours. Staining was performed as described in Materials and Methods, using antibodies directed against lambda light chain (red) and PDI as an ER marker (green) in **A**. and RCAS1 as a Golgi marker (green) in **B**. DAPI was used for nuclear staining (blue). Colocalization was determined using the RGB plot profile tool in ImageJ and indicated by similarity of the patterns of red and green peaks. Scale bar 10μm.

We have previously shown that the IBP inhibitor lovastatin causes an accumulation of lambda light chain in the ER in myeloma cells. [1] To further explore the effects of IBP inhibitors as well as bafilomycin A1 on light chain localization and trafficking, immunofluorescence studies were performed to assess ER and Golgi co-localization. Consistent with the ELISA studies, there was increased light chain staining in the lovastatin- and 3-PEHPC-treated cells but not in the bafilomycin A1-treated cells and the majority of the light chain co-localized with the ER marker PDI (Figure 5A). It should be noted that in untreated cells there was a peak of perinuclear fluorescence intensity that did not correlate with the ER. This focus of light chain was found to co-localize with RCAS1, a marker for the Golgi apparatus (Figure 5B). This staining pattern was lost in some of the IBP inhibitor-treated cells consistent with a block in trafficking from the ER to the Golgi. A common phenotype associated with disruption of the early secretory pathway and Rab GTPase function is the fragmentation of the Golgi apparatus. [27, 28] Indeed, many of the cells treated with IBP inhibitors exhibited a fragmented Golgi. Bafilomycin A1-treated cells, however, did not appear to have any significant Golgi fragmentation.

### Light chain is not a substrate for non-ER degradative pathways

**Figure 6:**
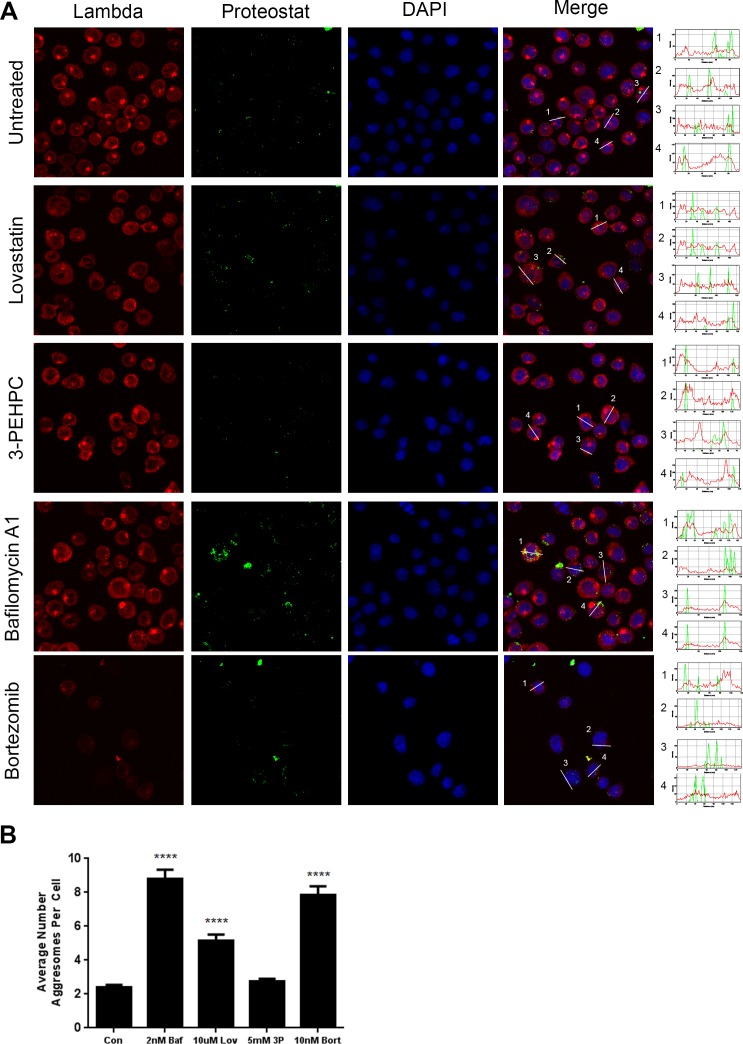
Light chain does not colocalize with aggresomes following IBP inhibitor treatment **A**. RPMI-8226 cells were incubated with 2 nM bafilomycin A1 (*Baf*), 10 μM lovastatin (*Lov*), or 5 mM 3-PEHPC (*3P*) for 48 h or 10 nM bortezomib for 24 h. Staining was performed as described in Materials and Methods, using Proteostat (Enzo Life Sciences, Inc.) (green) to visualize aggresomes and an antibody directed against lambda light chain (red). DAPI was used for nuclear staining (blue). Colocalization was determined using the RGB plot profile tool in ImageJ. Scale bar 10 μm. **B**. The average number of aggresomes per cell was quantified using the GFP-LC3 macro for ImageJ (mean ± SEM). **** denotes *P*<0.0001 from unpaired two-tailed *t-*test comparing treated with control cells.

We next investigated whether non-ER degradative pathways are involved in clearing monoclonal protein. No co-localization of the light chain was observed with aggresomes (Figure 6A) in either lovastatin- or 3-PEHPC-treated cells. Interestingly, we observed a marked increase in the average number of aggresomes per cell with lovastatin (Figure 6B) suggesting this treatment may be disrupting or overwhelming the ubiquitin proteasome system (UPS). Lovastatin has previously been reported to act a proteasome inhibitor when in its pro-drug, β-lactone ring form, [29] however more recent evidence has refuted this theory, [30, 31] making it unlikely that the increase in aggresome formation observed with lovastatin treatment is due to off- target global proteasome inhibition. However, while treatment with either bafilomycin A1 or the proteasome inhibitor bortezomib resulted in expected increases in aggresomes, [32] 3-PEHPC did not significantly increase aggresome numbers (Figure 6B). These results indicate that lovastatin-induced changes in aggresome numbers are not simply a consequence of disruption of Rab geranylgeranylation and light chain trafficking.

**Figure 7:**
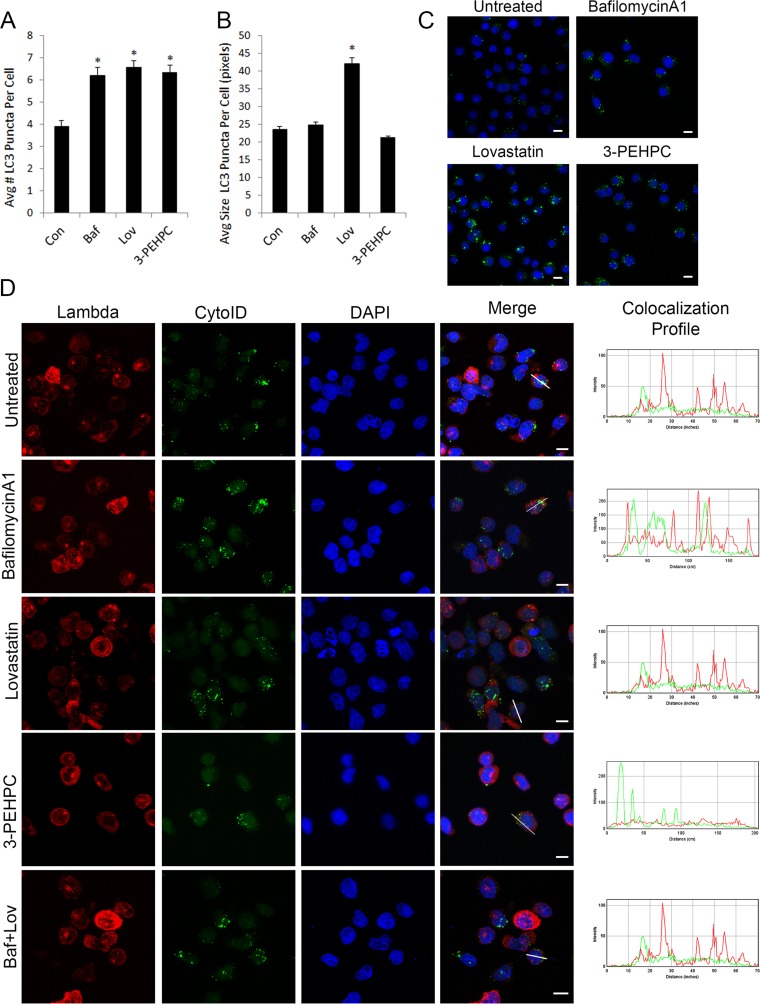
IBP inhibitors increase autophagosome numbers but light chain does not co-localize with autophagosomes U266 cells were treated with 2 nM bafilomycin A1 (*Baf*), 10 μM lovastatin (*Lov*) or 10 mM 3-PEHPC for 48 hours before processing for staining with LC3. **A**. The average number of LC3 puncta per cell and **B**. the average size of each punctate structure in pixels was quantified using the GFP-LC3 macro for ImageJ (mean ± SEM) (n>175). The **** denotes *p*<0.0001 from unpaired two-tailed *t-*test comparing treated with control cells. **C**. Representative confocal maximum projections are shown for each treatment with LC3 in green and DAPI staining the nucleus in blue. **D**. RPMI-8226 cells were incubated with 2 nM bafilomycin A1, 10μM lovastatin, 5mM 3-PEHPC or a co-treatment with bafilomycin A1 (*Baf*) and lovastatin (*Lov*) for 48 hours. Staining was performed as described in Materials and Methods, using CytoID (Enzo Life Sciences, Inc., Farmingdale, NY) to visualize autophagosomes and an antibody directed against lambda light chain. DAPI was used for nuclear staining. Colocalization was determined using the RGB plot profile tool in ImageJ. Scale bar 10μm.

Finally, we investigated the effects of the IBP inhibitors on autophagosomes and determined whether light chain co-localizes with autophagosomes. The increases in LC3-II observed by immunoblot correlated with an increase in the number of autophagosomes per cell (Figure 7A). Using immunofluorescence staining against LC3 and the ImageJ macro GFP-LC3 [33, 34] we detected a statistically significant increase (*P* < 0.0001) in the average number of LC3-positive puncta per cell when treated with lovastatin, 3-PEHPC or the positive control bafilomycin A1 (Baf) in U266 cells (6.57 ± 0.31, *n* = 250 ; 6.34 ± 0.33, *n* = 228; 6.21 ± 0.39 *n* = 175; mean ± SEM) when compared to untreated cells (3.9 ± 0.28, *n* = 235). While the drug treatments increased the number of autophagosomes per cell, the extent of the increase did not appear to correlate with the immunoblot data. We therefore examined autophagosome size as a possible explanation for this discrepancy using the same GFP-LC3 macro. Indeed, while bafilomycin A1-, 3-PEHPC- and untreated autophagosomes had a similar average size, the autophagosomes of cells treated with lovastatin were twice the size (Figure 7B). Figure 7C shows representative images used for the quantification.

Co-localization studies were then performed to determine whether lambda light chain accumulates in autophagosomes after IBP inhibitor treatment. Under control conditions, no co-localization of light chain with a marker for autophagosomes (CytoID) [35, 36] was observed, suggesting that under basal conditions, autophagy does not play a major role in the degradation of light chain (Figure 7D). In addition, when the fusion of autophagosomes with lysosomes was blocked using bafilomycin A1, there was no co-localization of light chain with autophagosome vesicles. Notably, treatment with neither lovastatin nor 3-PEHPC caused co-localization of lambda light chain with the CytoID stain (Figure 7D). Additionally, utilizing bafilomycin A to block the fusion of autophagosomes with lysosomes in combination with lovastatin did not cause accumulation of light chain in autophagosomes, suggesting the observed absence of co-localization is not due to autophagic degradation of light chain after lovastatin treatment. Furthermore, light chain did not accumulate in LAMP-1 positive lysosomal vesicles under any of these conditions (Supplementary Figure 11). In aggregate these results indicate that the block in light chain trafficking and subsequent intracellular accumulation induced by IBP inhibitors cannot be rescued by alternative cellular degradative pathways.

## DISCUSSION

We have previously demonstrated that agents which interfere with geranylgeranylation of Rab GTPases cause disruption of monoclonal protein trafficking in myeloma cells, leading to ER stress and apoptosis. [1, 2] Whether this accumulated monoclonal protein is a trigger for induction of autophagy and whether autophagy represents a mechanism by which myeloma cells can degrade the aggregated protein have been unanswered questions. Previous studies have reported mixed conclusions regarding the effects of IBP inhibitors on autophagy and the roles that isoprenoids and/or prenylated proteins play in regulating autophagy. [16-20], [37-39] In the present studies we demonstrate that the effects of agents which indirectly disrupt protein prenylation by altering isoprenoid levels are distinct from those that directly inhibit protein prenylation. In particular, these are the first reported studies to utilize a specific GGTase II inhibitor and thus directly test the previously raised hypothesis that inhibition of GGTase II substrate function induces autophagy. [20]

Three agents were used in these studies to disrupt Rab geranylgeranylation: lovastatin, DGBP, and 3-PEHPC. That each of these agents disrupts Rab geranylgeranylation in different ways has allowed us to differentiate between the roles of prenylated proteins and the isoprenoid pathway in regulating autophagy. Treatment with lovastatin disrupts all protein prenylation as well as depletes cells of all isoprenoids downstream of mevalonate (Figure 1, Supplementary Figure 2). DGBP, by virtue of the fact that it targets the pathway more downstream, selectively disrupts protein geranylgeranylation and depletes cells of isoprenoids derived from GGPP. 3-PEHPC directly inhibits GGTase II and does not impact isoprenoid levels. We hypothesized that these agents could influence autophagy in a number of ways including: 1) induction as a consequence of isoprenoid depletion, 2) induction as a consequence of aggregated monoclonal protein, 3) inhibition as a consequence of disruption of geranylgeranylation of Rabs known to be involved in autophagic vesicles, or 4) inhibition or induction as a consequence of disruption of protein farnesylation or geranylgeranylation. Thus depending on the extent to which an agent affects each of these processes there could be varying degrees of both induction and inhibition of autophagy.

That treatment with lovastatin, DGBP, or 3-PEHPC resulted in similar levels of intracellular light chain (Supplementary Figure 6) yet induced different levels of LC3-II (Figure 2C) suggests that the accumulated light chain by itself is insufficient as an explanation for changes in autophagic processes. Furthermore, that the combination of specific GGTase I and GGTase II inhibitors does not recapitulate the effects of lovastatin or DGBP (Figure 2C) suggests that factors aside from protein geranylgeranylation influence autophagy. We had predicted that if the changes in LC3-II and p62 levels induced by lovastatin were dependent on disruption of protein geranylgeranylation, then co-incubation with mevalonate or GGPP, but not FPP, would prevent those effects. As predicted, both mevalonate and GGPP completely prevented lovastatin's effects. However, despite the inability of FPP to rescue protein geranylgeranylation (Figures 2B, Supplementary Figure 4), its addition did partially prevent the lovastatin-induced increase in LC3-II (Figure 2C, Supplementary Figure 5) and decrease in p62 (Figure 3B). The studies utilizing zaragozic acid A (Figure 2D) provided further evidence that depletion of non-sterol isoprenoids is important in regulating autophagic flux. Wasko et al. [20] concluded that because DGBP increased LC3-II levels but a GGTase I inhibitor did not, that the effects of DGBP were due to disruption of Rab geranylgeranylation. However, our studies with 3-PEHPC (alone or in combination with the GGTase I inhibitor) provide evidence that the effects of DGBP are not due solely to disruption of Rab geranylgeranylation. In aggregate these data demonstrate that in myeloma cells, perturbation of the IBP upstream of GGPP is a trigger for autophagy.

The decrease in levels of Atg3 in lovastatin-treated cells is noteworthy (Figure 3). Atg3 is part of the Atg8, Atg7 ubiquitin-like conjugation system and is involved in the conjugation of phosphatidylethanolamine (PE) to Atg8. [40] Relatively little is known about the regulation of Atg3 protein levels, however it has been shown to be a substrate for caspase-8 following activation of the extrinsic apoptotic pathway. [41] It is also intriguing that lovastatin causes an increase in the average size of the autophagic vesicles in addition to an increase in the number of puncta (Figure 7). The mechanisms that regulate autophagosome size have not yet been fully delineated, although a role for Atg8 has been implicated. [42] These results warrant further investigation into how disruption of isoprenoid levels may affect Atg3 regulation and autophagosome size and exploration of whether they are functionally linked.

Autophagy appears to play a pro-survival role in both healthy and malignant plasma cells by regulating immunoglobulin production [43] however it is not clear if this is due to a signaling mechanism or because immunoglobulin is an autophagic substrate. We hypothesized that if light chain is a substrate for autophagy in myeloma cells then it would be co-localized with autophagic vesicles. However, our studies did not reveal accumulation of light chain in autophagosomes under any treatment condition (Figure 7), suggesting that autophagy is not directly involved in the clearance of light chain. It is possible that if the light chain accumulated in autophagosomes is misfolded or aggregated it may obscure the epitope recognized by the antibody used in the microscopy studies, thus resulting in a false negative. However, blocking autophagic flux with protease inhibitors did not alter intracellular light chain levels as measured by ELISA (Figure 3E). Finally, the co-treatment studies with the IBP and autophagy inhibitors suggest that the late stages of autophagy are critically important for myeloma cell survival after IBP inhibition. These results are similar to studies performed in malignant glioma cells in which inhibition of early stages of autophagy diminished the cytotoxic effects of chemotherapy agents while inhibition of late stages of autophagy significantly enhanced the cytotoxic effects. [44-46] The mechanisms underlying these observations have not been fully elucidated but are likely a consequence of the many connections between the autophagy and apoptosis pathways. [47]

In summary, we have demonstrated that the effects of agents which disrupt isoprenoid biosynthesis are distinct from direct geranylgeranyl transferase inhibition such that depletion of non-sterol isoprenoids downstream of FPP induces autophagy while direct inhibition of Rab GGTase results in a net block in autophagy. This distinction is important as it not only provides new insight into the role of isoprenoids in regulating autophagy, but it also reveals that GGTase II inhibition in myeloma cells does not induce a self-protective mechanism which must be overcome in order to maximize the cytotoxic effects. These studies, in conjunction with our finding that myeloma cells cannot degrade accumulated light chain via autophagosomes/lysosomes or aggresomes, support further development of specific GGTase II inhibitors as anti-myeloma agents.

## MATERIALS AND METHODS

### Reagents

Lovastatin (M2147), mevalonolactone (M4667, converted to mevalonate prior to use), farnesyl pyrophosphate (F6892), geranylgeranyl pyrophosphate (G6025), bafilomycin A1 (B1793), 3-methyladenine (M9281), E64d (E8640), pepstatin A (P5318), and zaragozic acid A (Z2626) were obtained from Sigma. GGTI-2133 (sc-221668) was purchased from Santa Cruz Biotechnology. Bortezomib (2204) was obtained from Cell Signaling Technology. Digeranyl bisphosphonate (DGBP) [48] and 3-PEHPC [23] were kindly provided by Professor David Wiemer, Department of Chemistry, University of Iowa.

### Cell culture

Human myeloma cell lines (RPMI-8226, U266, MM.1S) were purchased from American Type Culture Collection (ATCC) (Manassas, VA). Cells were grown in media (per ATCC specifications) supplemented with heat-inactivated fetal bovine serum (FBS), glutamine and penicillin-streptomycin at 37°C and 5% CO_2_. ALMC-2 cells were obtained from Dr. Diane Jelinek, Mayo Clinic (Rochester, MN) and were grown in Iscove modified Dubelcco medium supplemented with FBS, glutamine, penicillin-streptomycin, IL-6, and IGF-1. [49]

### Monoclonal protein quantitation

Cells were incubated in the presence or absence of drugs for specified periods of time. The cells were lysed in RIPA buffer (0.15M NaCl, 1% sodium deoxycholate, 0.1% SDS, 1% Triton (v/v) X-100, 0.05 M Tris HCl, pH 7.4) containing protease and phosphatase inhibitors. Protein content was determined using the bicinchoninic acid (BCA) method. A human lambda light chain kit (E80-116, Bethyl Laboratories) was used to quantify intracellular monoclonal protein levels.

### Immunoblotting

Following incubation with drugs, cells were collected, washed with PBS, and lysed in RIPA buffer as described above. Protein content was determined using the BCA method. Equivalent amounts of cell lysate were resolved by SDS-PAGE, transferred to polyvinylidene difluoride membrane, probed with the appropriate primary antibodies, and detected using HRP-linked secondary antibodies and Amersham Pharmacia Biotech ECL Western blotting reagents per manufacturer's protocols. For Rab6, cells were lysed with Triton X-114 to generate detergent (membrane) fractions. [20] Supplementary Table 1 details the primary and secondary antibodies.

### Measurement of intracellular FPP and GGPP levels

Intracellular FPP and GGPP levels were measured using the previously reported reversed phase HPLC methodology. [50] Briefly, following incubation with drugs, cells were collected and counted using Trypan blue staining and a Bio-Rad TC10 automated cell counter. Isoprenoid pyrophosphates were extracted from cell pellets with extraction solvent (butanol /75 mM ammonium hydroxide/ethanol 1:1.25:2.75). After drying down by nitrogen gas, the FPP and GGPP in the residue were incorporated into fluorescently labeled GCVLS or GCVLL peptides (Biosynthesis, Inc.) by recombinant FTase or GGTase I, respectively (Jena Biosciences). The prenylated fluorescent peptides were separated and quantified by reversed phase HPLC with fluorescence detection.

### Annexin V/propidium iodide flow cytometry

Following incubation with drugs, 5 × 10^5^ cells were spun down and resuspended in 0.5mL HEPES solution (10mM HEPES, 150mM NaCl, 1mM MgCl_2_, 5mM KCl, 1.8mM CaCl_2_). Cells were incubated with 5μl FITC annexin V (Life Technologies) for 10 minutes at room temperature followed by addition of 10μl of a 50μg/ml solution of propidium iodide (Life Technologies). Flow cytometry was performed using a BD LSR II Flower Cytometer at the Flow and Image Cytometry Core facility at the Roswell Park Cancer Institute. Data was analyzed using WinList 3D 8.0 software (Verity Software House, Topsham, ME).

### MTT assay

Cells were seeded (2.5 × 10^4^ cells/100 μL per well) in 96-well flat-bottom plates and incubated with drugs for 48 hours. The MTT assay was performed as previously described. [51] The absorbance for control cells treated with solvent only was defined as an MTT activity of 100%.

### Immunofluorescence microscopy studies

RPMI-8226 cells were incubated for 48 hours in the absence or presence of drugs and were plated on poly-l-lysine-coated coverslips for processing. The coverslips were then washed with PBS and cells were fixed with 4% formaldehyde, permeabilized in 0.1% Triton X-100 and blocked with 4% goat serum in PBS. For LC3 staining, cells were permeabilized with 0.1% saponin. The coverslips were incubated with primary antibodies for 1 hour, washed with PBS, and then incubated with secondary antibodies for 30 minutes. The coverslips were again washed with PBS and mounted in Vecta-Shield with DAPI. Supplementary Table 2 details the primary and secondary antibodies. Autophagosome and aggresome staining were performed using CYTO-ID® Autophagy (1:100) and PROTEOSTAT® Aggresome (1:2000) detection kits according to manufacturers' instructions (Enzo Life Sciences, Inc., Farmingdale, NY). [35, 36] Microscopy was performed using a Leica TCS SP2 spectral confocal microscope with a 63× objective at the Flow and Image Cytometry Core facility at the Roswell Park Cancer Institute. Images were processed using ImageJ software

### Statistics

Two-tailed *t-*testing was used to calculate statistical significance. An α of 0.05 was set as the level of significance. Combination indices for the MTT assays were determined via CalcuSyn software (Biosoft). The software analyzes drug interactions based on the method of Chou and Talalay. [52]

## SUPPLEMENTARY MATERIAL FIGURES AND TABLES


