# Supplementary material for: Mechanisms for autophagy modulation by isoprenoid biosynthetic pathway inhibitors in multiple myeloma cells
Source: Oncotarget. 2015 Nov 22;6(39):41535–49. doi: 10.18632/oncotarget.6365 (PMC4747172; doi:10.18632/oncotarget.6365)
Supplement: Supplementary file 1 [file oncotarget-06-41535-s001.pdf]

Mechanisms for autophagy modulation by isoprenoid biosynthetic pathway inhibitors in multiple myeloma cells

Supplementary Material

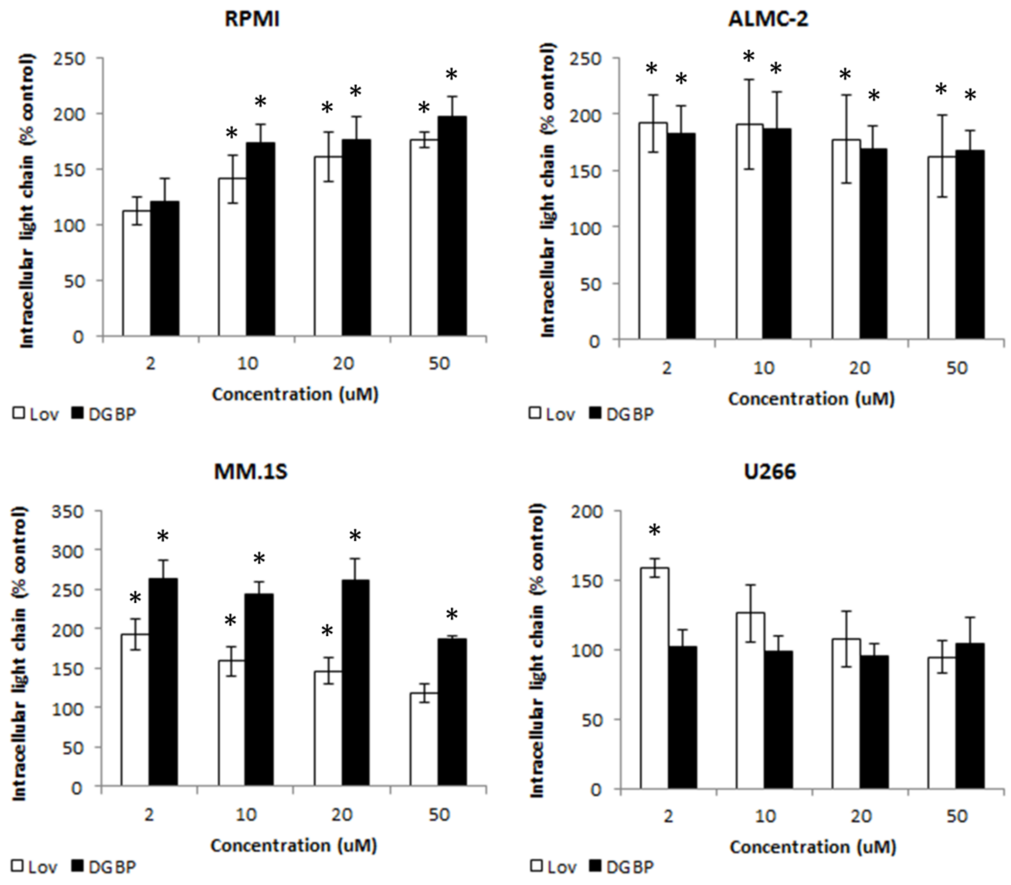

Supplementary Figure 1. Effects of IBP inhibitors on intracellular light chain levels. Intracellular lambda light chain levels were measured via ELISA. Data are expressed as a percentage of control (mean ± standard deviation of 3 independent experiments). The * denotes $p<0.05$ per two-sided t-testing comparing treated cells to untreated (control) cells. Cells were incubated for 48 hrs in the presence or absence of lovastatin (*Lov*, 2-50 μM) or DGBP (2-50 μM).

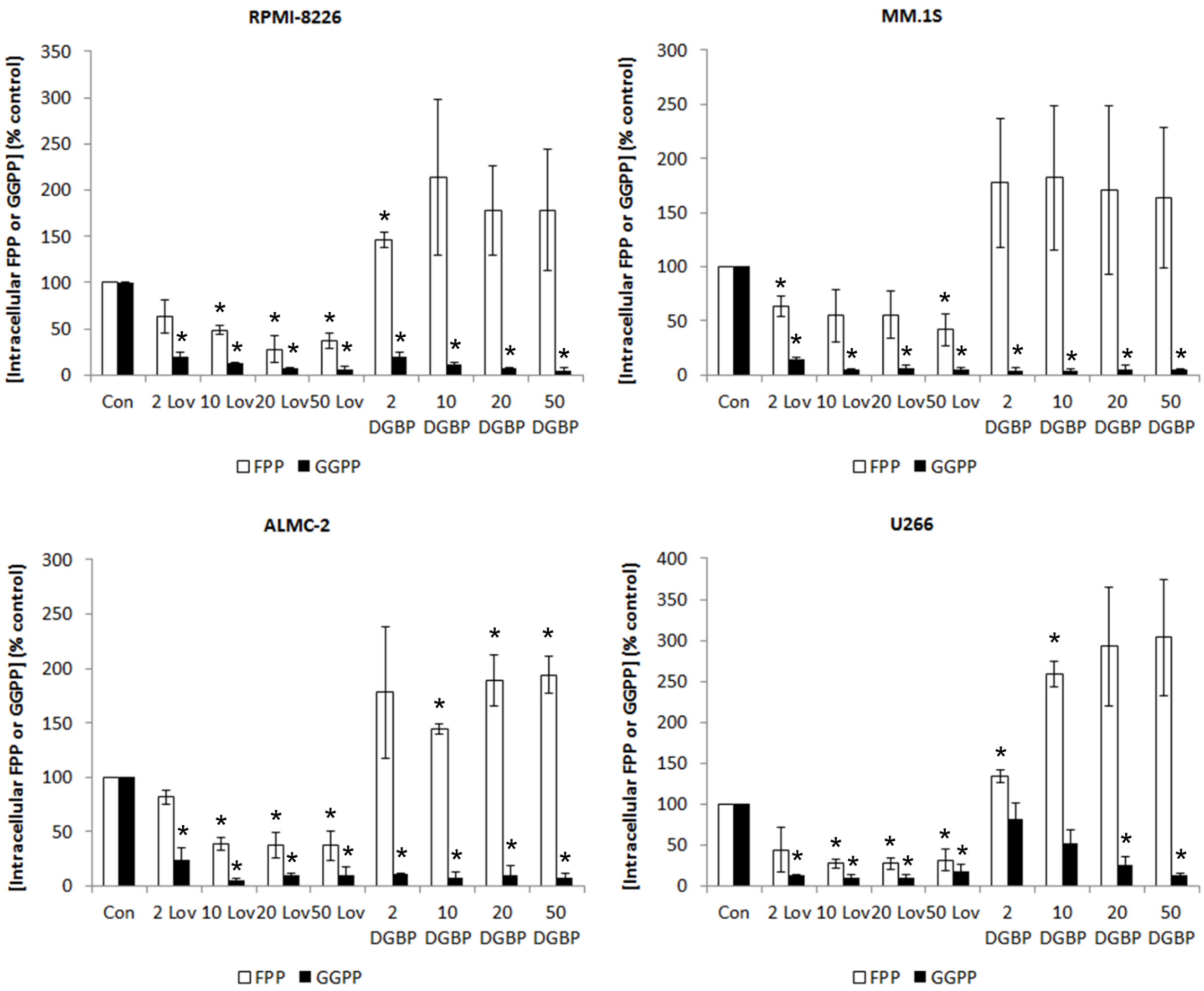

Supplementary Figure 2. Effects of IBP inhibitors on intracellular FPP and GGPP levels. Cells were incubated for 48 hrs in the presence or absence of lovastatin (*Lov*, 2-50 μM) or DGBP (2-50 μM). FPP and GGPP were subsequently extracted and quantified as described in the Materials and Methods section. Data are expressed as a percentage of control (mean ± standard deviation of 2 independent experiments). The * denotes $p<0.05$ per two-sided t-testing comparing treated cells to untreated (control) cells.

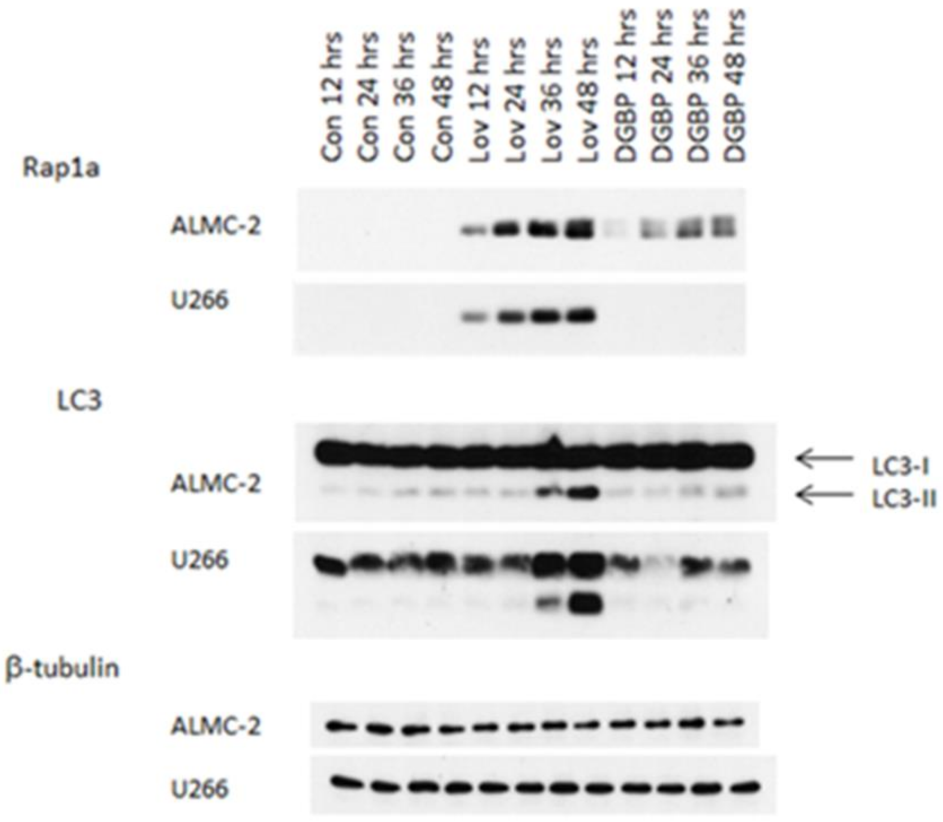

Supplementary Figure 3. Time-dependent effects of IBP inhibitors on protein geranylgeranylation and LC3-II levels. Cells were incubated for 12 to 48 hours in the presence or absence of lovastatin (*Lov*, 10 μM) or DGBP (10 μM). Immunoblot analysis of unmodified Rap1a, LC3, and b-tubulin.

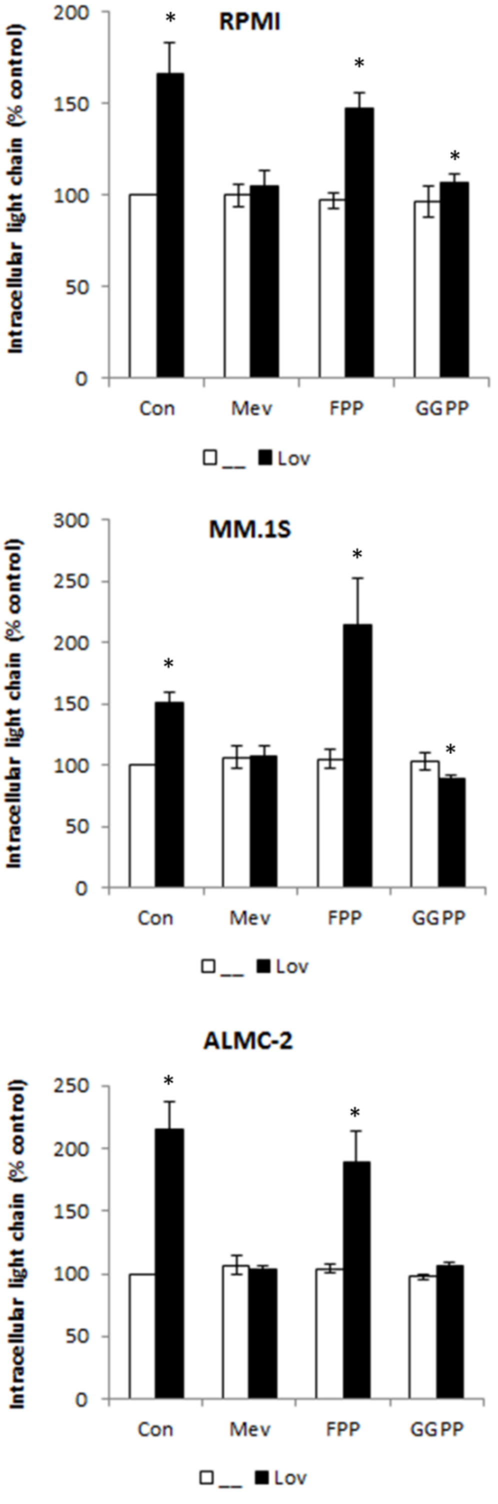

Supplementary Figure 4. Mevalonate and GGPP, but not FPP, prevents lovastatin-induced changes in intracellular light chain levels. Cells were incubated for 48 hrs in the

presence of 10 μM lovastatin (*Lov*) and/or mevalonate (*Mev*, 5 mM), FPP (10 μM), or GGPP (10 μM) for 48 hrs. Intracellular lambda light chain levels were measured via ELISA. Data are expressed as a percentage of control (mean ± standard deviation of 3 independent experiments). The * denotes $p<0.05$ per two-sided t-testing comparing treated cells to untreated (control) cells.

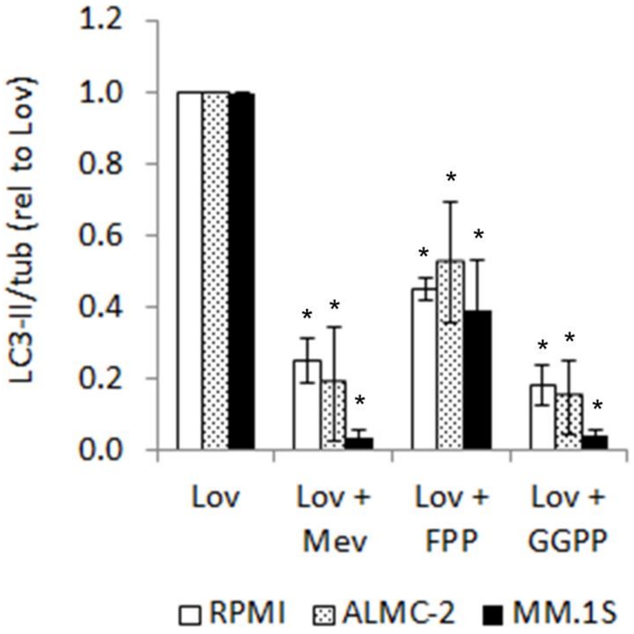

Supplementary Figure 5. Densitometric analysis of LC3-II levels (normalized to β-tubulin levels) for the combination treatments normalized to lovastatin alone is shown. Data are displayed as average +/- standard deviation (n=3 independent experiments). * denotes p-value <0.05 from two-sided t-testing.

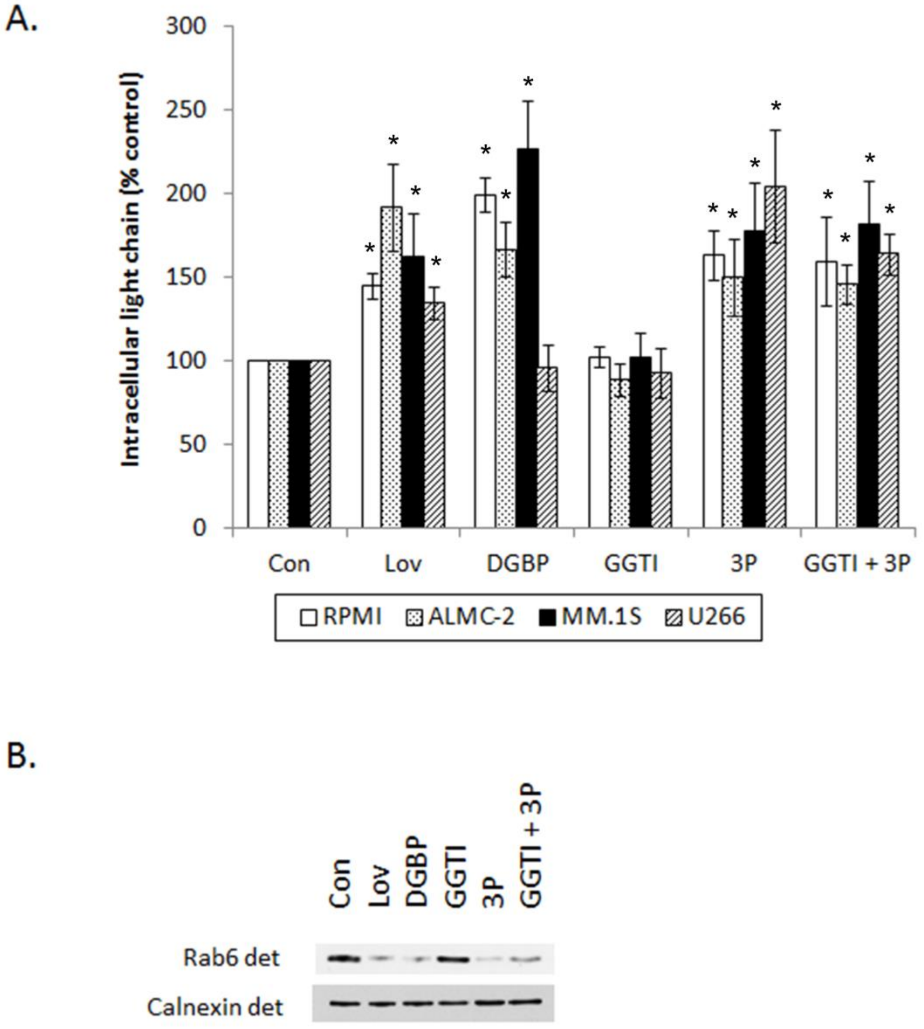

Supplementary Figure 6. Agents which deplete cells of GGPP or directly inhibit GGTase II increase intracellular light chain levels. A) Cells were incubated for 48 hrs in the presence or absence of 10 μM lovastatin (*Lov*), 10 μM DGBP, 10 μM GGTI-2133 (*GGTI*), 10 mM 3-PEHPC (*3P*), or the combination of 10 μM GGTI-2133 and 10 mM 3-PEHPC (*GGTI + 3P*). Intracellular lambda light chain levels were measured via ELISA. Data are expressed as a percentage of control (mean ± standard deviation of 3 independent experiments). The * denotes $p<0.05$ per two-sided t-testing comparing treated cells to untreated (control) cells. **B).** RPMI-8226 cells were incubated as in A). Cells were lysed with Triton X-114 and immunoblot analysis of Rab6 and calnexin (as a loading control) was performed on the detergent fractions to assess membrane-bound (prenylated) Rab6 levels.

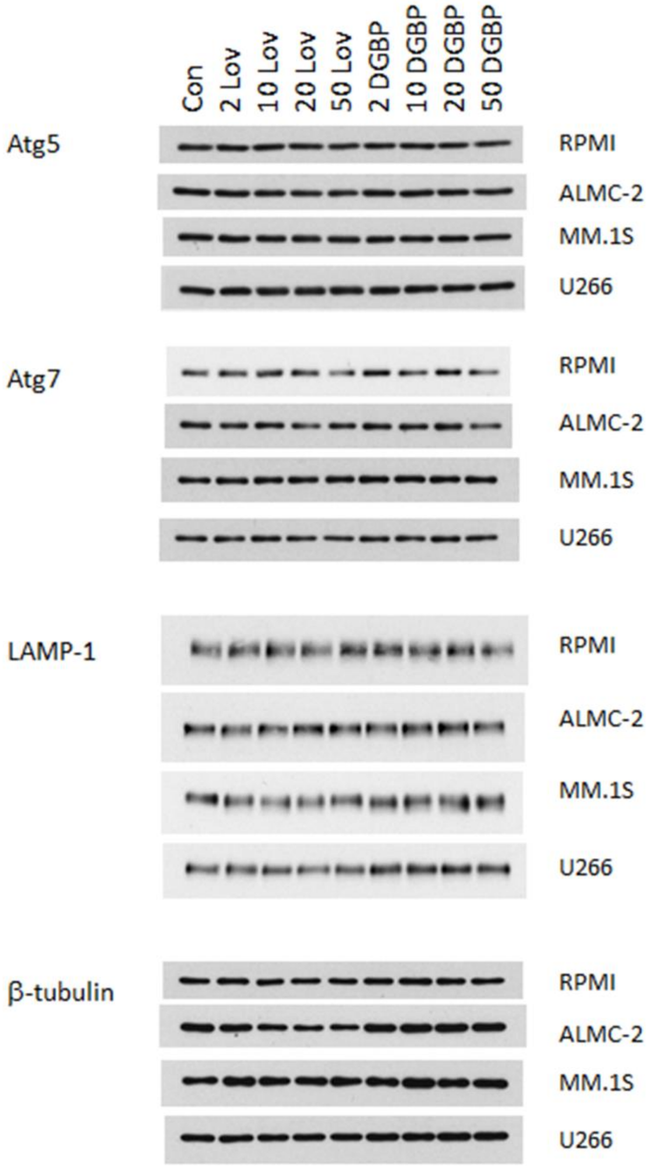

Supplementary Figure 7. Atg5, Atg7, and LAMP-1 levels are not altered by lovastatin or DGBP. Cells were incubated for 48 hours in the presence or absence of lovastatin (*Lov*, 2-50 μM) or DGBP (2-50 μM). Immunoblot analysis of Atg5, Atg7, LAMP-1, and β-tubulin (as a loading control) is shown. Blots are representative of three independent experiments.

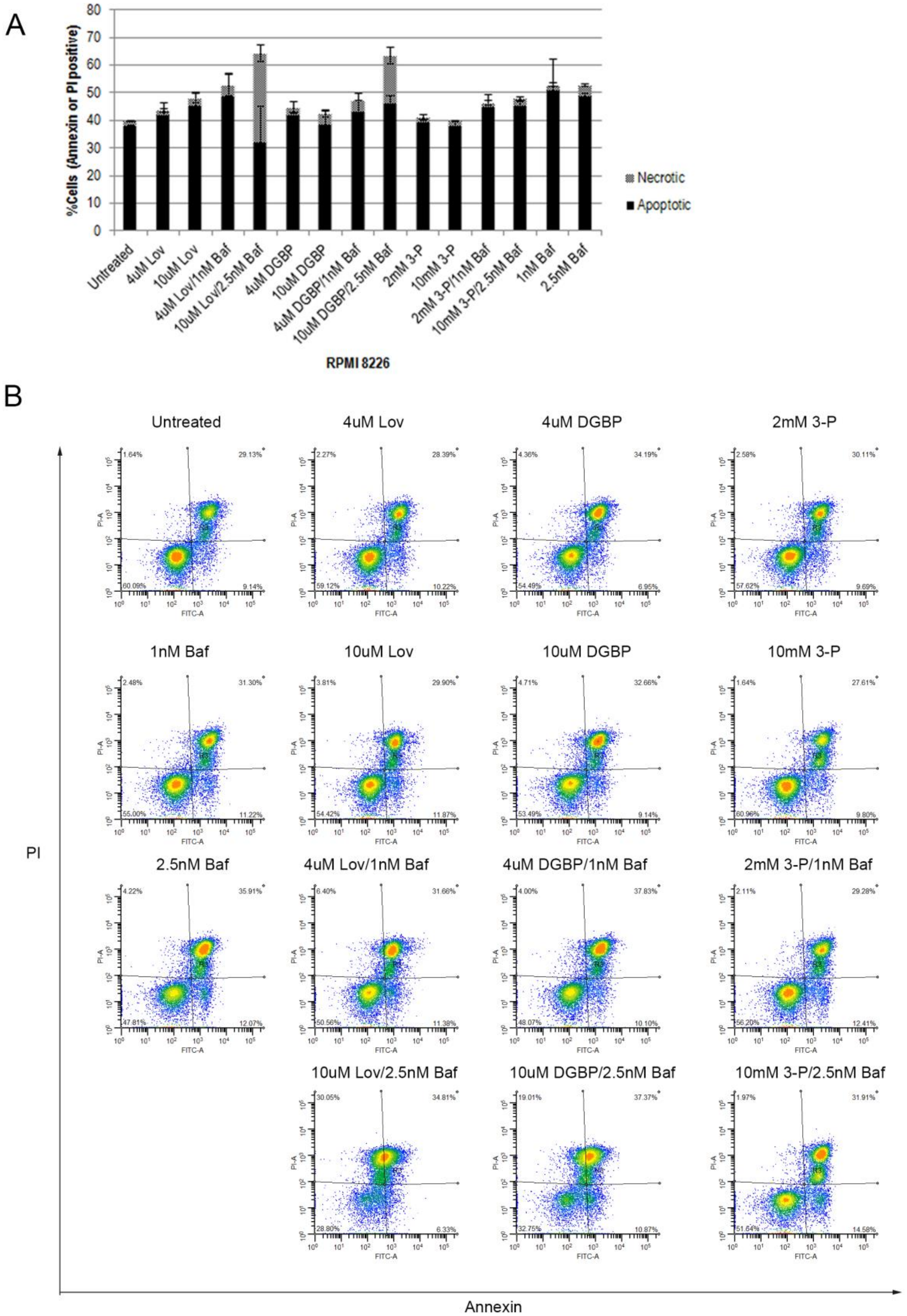
A
%Cells (Annexin or PI positive)
Necrotic
Apoptotic
Untreated
4uM Lov
10uM Lov
4uM Lov/1nM Baf
10uM Lov/2.5nM Baf
4uM DGBP
10uM DGBP
4uM DGBP/1nM Baf
10uM DGBP/2.5nM Baf
2mM 3-P
10mM 3-P
2mM 3-P/1nM Baf
10mM 3-P/2.5nM Baf
1nM Baf
2.5nM Baf
RPMI 8226
B
Untreated
4uM Lov
4uM DGBP
2mM 3-P
1nM Baf
10uM Lov
10uM DGBP
10mM 3-P
2.5nM Baf
4uM Lov/1nM Baf
4uM DGBP/1nM Baf
2mM 3-P/1nM Baf
10uM Lov/2.5nM Baf
10uM DGBP/2.5nM Baf
10mM 3-P/2.5nM Baf
PI
Annexin

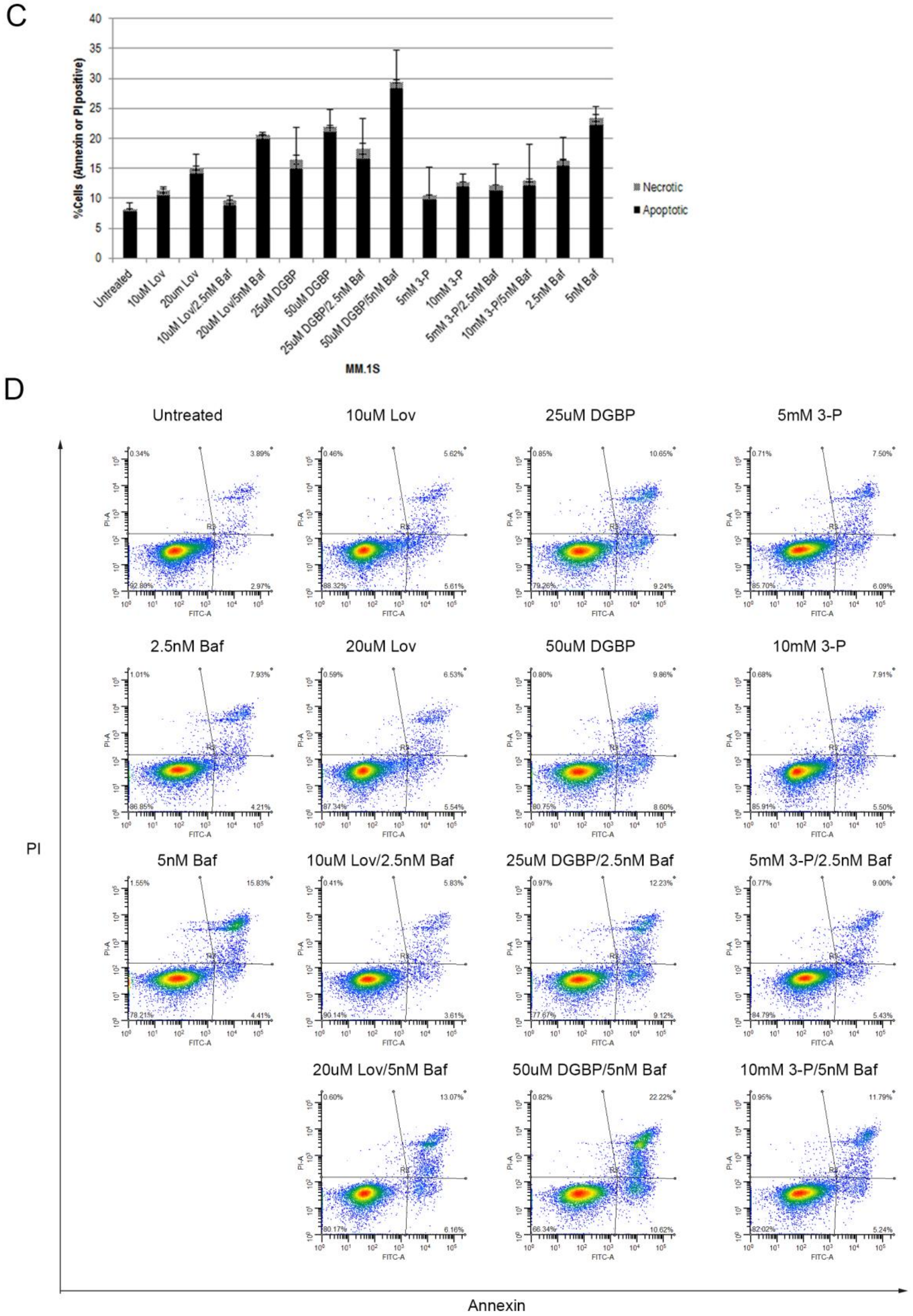

Supplemental Figure 8. Bafilomycin A1 enhances lovastatin and DGBP-mediated apoptosis. Annexin-V FITC/propidium iodide (PI) flow cytometry assays were performed in RPMI-8226 (A and B) and MM.1S cells (C and D) treated with bafilomycin A1 (*Baf*) and/or lovastatin (*Lov*), DGBP or 3-PEHPC (*3-P*) for 48 hours. Data in A and C is expressed as a percentage of cells positive for annexin and/or PI fluorescence.

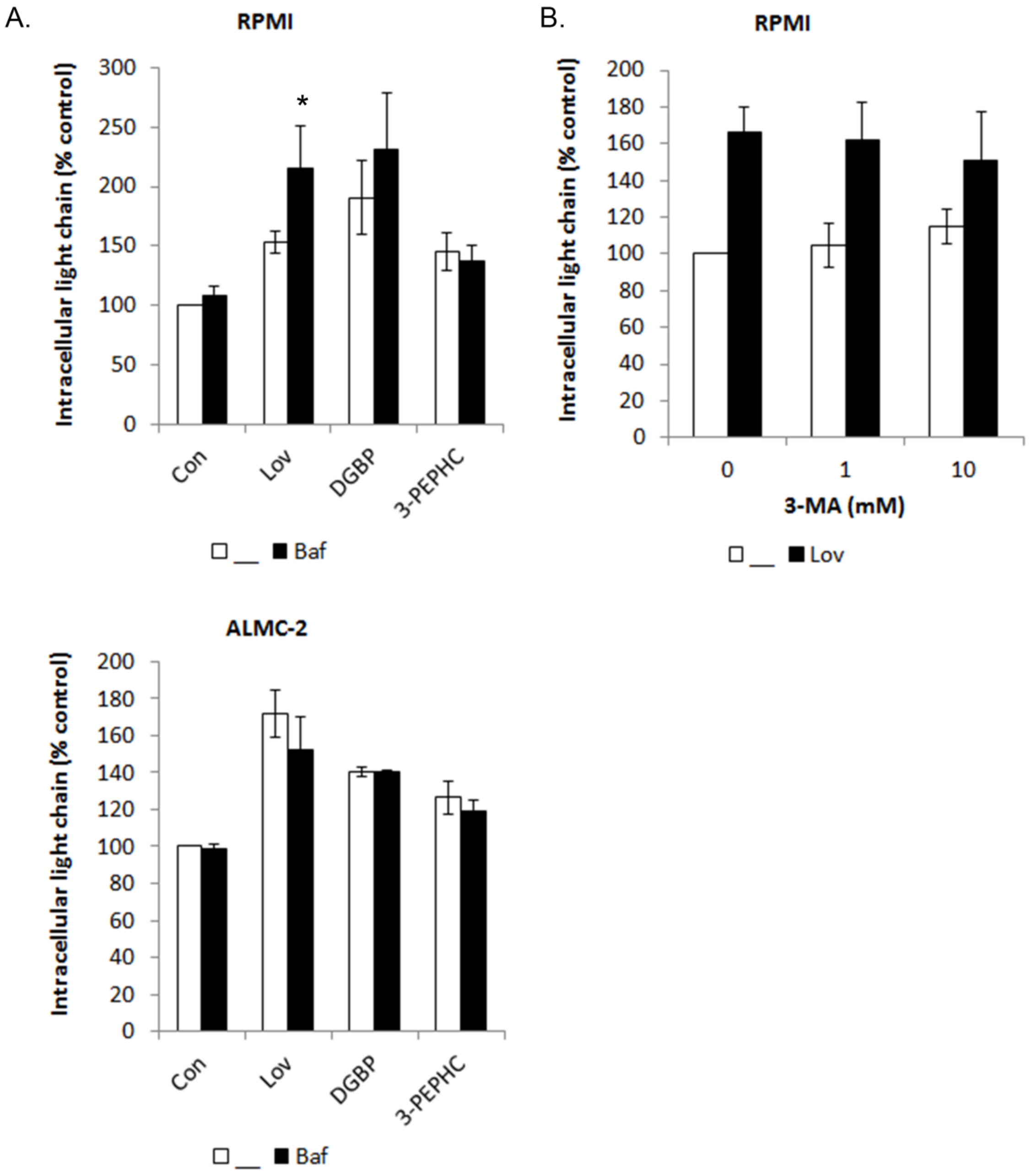

Supplementary Figure 9. Effects of autophagy inhibitors in combination with isoprenoid biosynthetic pathway inhibitors on intracellular light chain levels. Intracellular lambda light chain levels were measured via ELISA. Data are expressed as a percentage of control (mean $\pm$ standard deviation of 3 independent experiments). **A)** RPMI-8226 or ALMC-2 cells were incubated for 48 hours in the presence or absence of 10 μM lovastatin (*Lov*), 10 μM DGBP, 10 mM 3-PEHPC (*3P*) with or without 2 nM bafilomycin A1 (*Baf*). Two-sided t-testing was performed to compare the IBP inhibitor alone vs in combination with bafilomycin A1. **B)** RPMI-8226 cells were incubated for 48 hours in the presence or absence of 3-methyladenine (*3-MA*)

and 10 μM lovastatin (*Lov*). Two-sided t-testing was performed to compare lovastatin alone vs in combination with 3-MA. No significant differences between the treatment groups were observed.

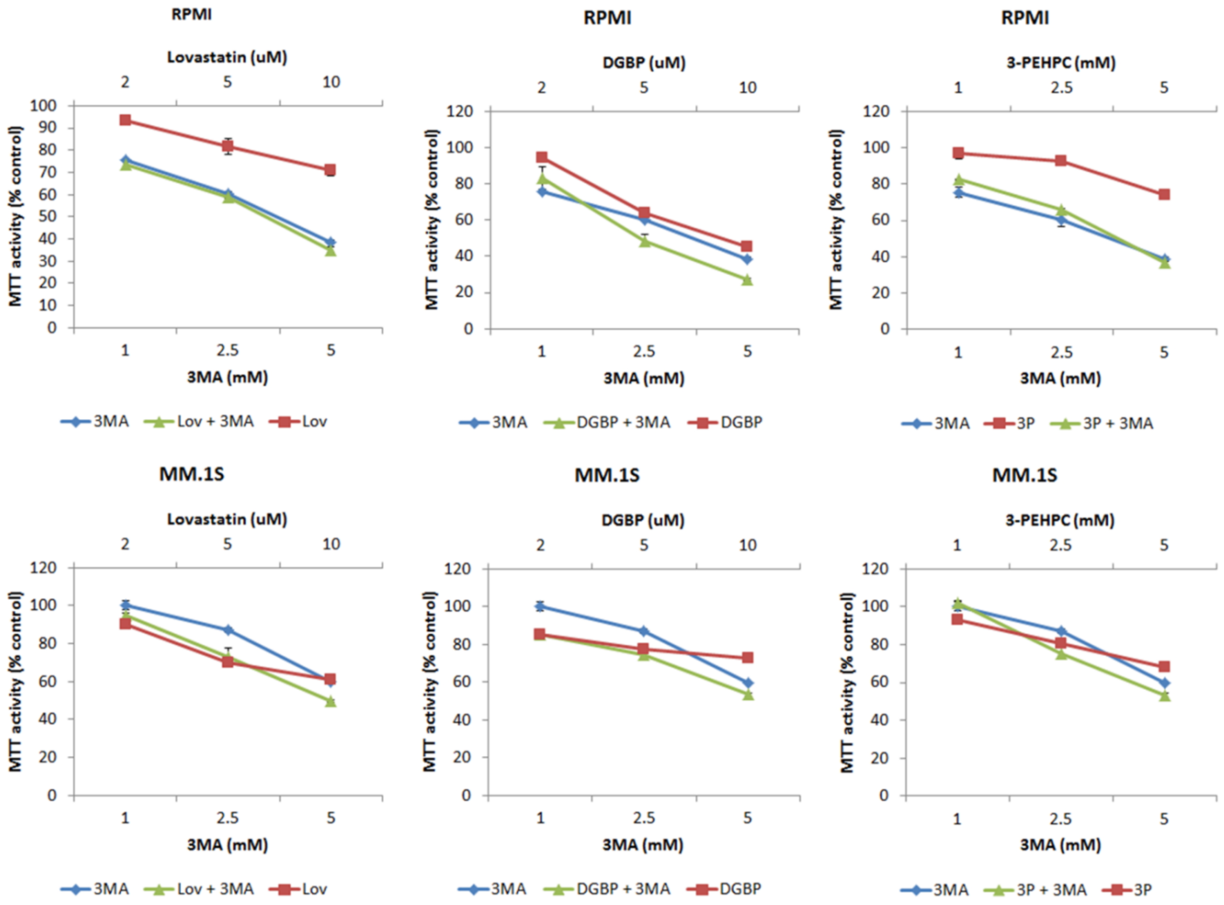

Supplementary Figure 10. 3-methyladenine does not increase isoprenoid biosynthetic pathway inhibitor-induced cytotoxicity. MTT cytotoxicity assays were performed with RPMI-8226 and MM.1S cells treated with 3-methyladenine (*3MA)* and/or lovastatin *(Lov*), DGBP, or 3-PEHPC (*3P*) for 48 hrs. Data are expressed as percentage of control (mean ± SD, n=4).

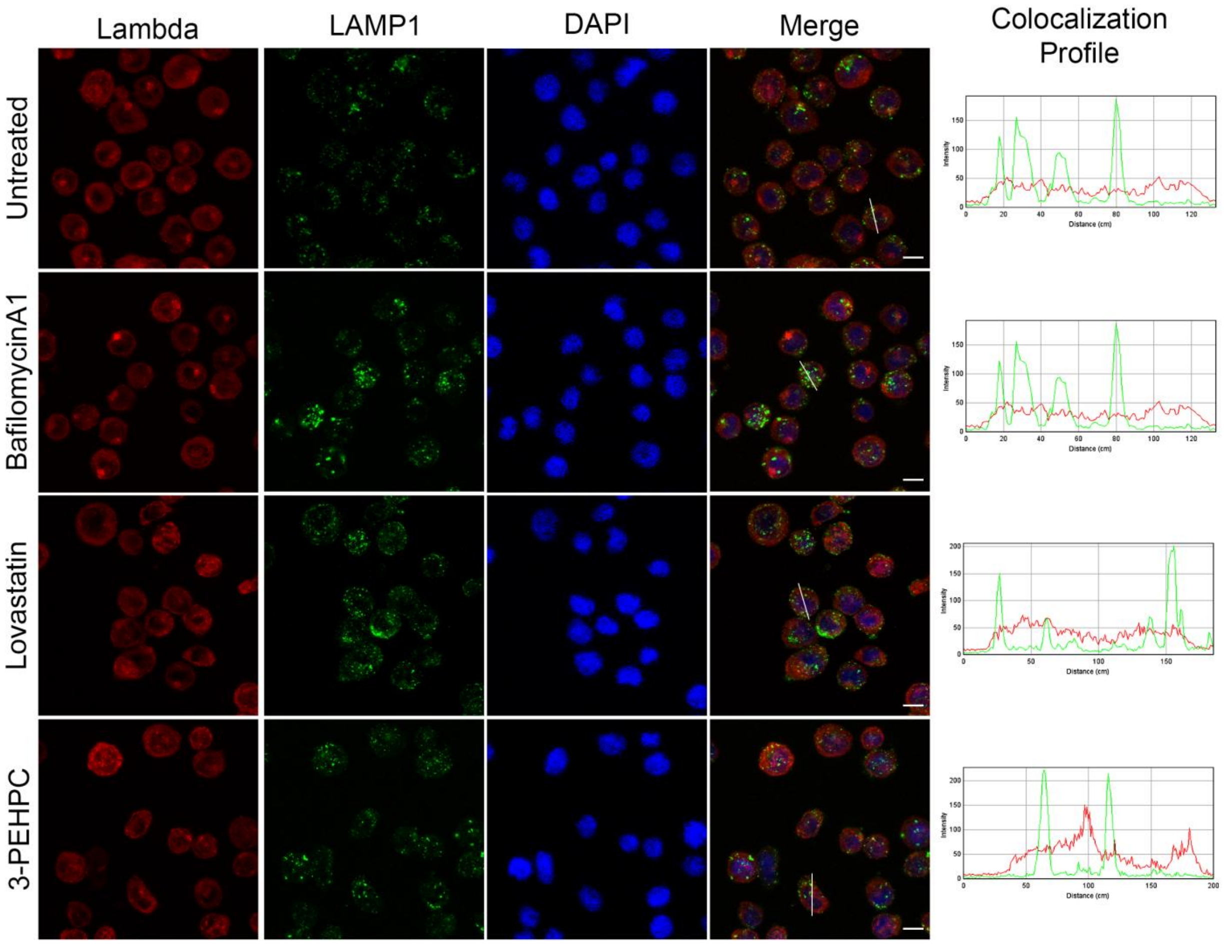

Supplementary Figure 11. Lambda light chain does not colocalize with lysosomes following IBP inhibitor treatment. RPMI-8226 cells were incubated with 2 nM bafilomycin A1, 10 µM lovastatin, or 5 mM 3-PEHPC for 48 h. Staining was performed as described in Materials and Methods, using antibodies directed against lambda light chain (red) and LAMP-1 as a lysosomal marker (green). DAPI was used for nuclear staining (blue). Colocalization was determined using the RGB plot profile tool in ImageJ and indicated by dissimilarity of the patterns of red and green peaks. Scale bar 10 µm.

Supplementary Table 1. Antibodies used for immunoblot analysis

	**Primary Antibody Source (catalog number) (dilution)**	**Secondary Antibody Source (catalog number) (dilution)**
Atg3	Cell Signaling Technology (3415P) (1:500)	Cell Signaling Technology (7074S) (1:1000)
Atg5	Cell Signaling Technology (12994S) (1:1000)	Amersham (NA934) (1:1000)
Atg7	Cell Signaling Technology (8558P) (1:250)	Amersham (NA934)* (1:1000)
Calnexin	Santa Cruz Biotechnology (sc-23954) (1:250)	Amersham (NA934) (1:1000)
Cleaved caspase 3	Cell Signaling Technology (9664S) (1:500)	Amersham (NA934) (1:1000)
Cleaved caspase 9	Cell Signaling Technology (9501S) (1:500)	Amersham (NA934) (1:1000)
LAMP-1	Cell Signaling Technology (3243S) (1:1000)	Amersham (NA934)* (1:2000)
LC3	MBL International Corporation (PM046) (1:1000)	Amersham (NA934) (1:1000)
p62	Cell Signaling Technology (8025S) (1:500)	Amersham (NA934)* (1:1000)
Rap1a	Santa Cruz Biotechnology (sc-1482) (1:200)	Santa Cruz Biotechnology (sc-2020) (1:1000)
Rab6	Santa Cruz Biotechnology (sc-310) (1:5000)	Amersham (NA934) (1:2000)
β-tubulin	Sigma (T5201) (1:40000)	Amersham (NA931) (1:1000)

*Secondary antibody source for ALMC-2 cells was Cell Signaling Technology (7074S)

Supplementary Table 2. Antibodies used for immunofluorescence analysis

	**Primary Antibody Source (catalog number) (dilution)**	**Secondary Antibody Source (catalog number) (dilution)**
PDI	Santa Cruz Biotechnology (sc-20132) (1:100)	Pierce DyLight® 488 goat anti-rabbit IgG (35552) (1:500)
Ig λ	Santa Cruz Biotechnology (sc-51638) (1:200)	Pierce DyLight® 633 goat anti-mouse IgG (35512) (1:500)
LC3	MBL International (PM036) (1:1000)	Pierce DyLight® 488 goat anti-rabbit IgG (35552) (1:500)
LAMP-1	Cell Signaling Technology (9091) (1:100)	Pierce DyLight® 488 goat anti-rabbit IgG (35552) (1:500)

Supplementary Table 3. Combination indices (CI) for MTT cytotoxicity assays. A synergistic interaction has a CI of <0.9, an additive interaction has a CI of 0.9-1.1, and an antagonistic interaction has a CI of >1.1.

	RPMI-8226[a]	MM.1S[b]
Baf + Lov	0.43	0.6
Baf + DGBP	0.67	0.2
Baf + 3P	1.11	0.84
3MA + Lov	1.16	1.71
3MA + DGBP	1.40	1.03
3MA + 3P	1.38	1.58

[a]Combination indices for ED_{50}.
[b]Combination indices for ED_{30}.
